# Editorial: Women in science: Public Health Education and Promotion 2022

**DOI:** 10.3389/fpubh.2023.1204113

**Published:** 2023-05-04

**Authors:** Rosemary M. Caron, Shazia Qasim Jamshed, Melody S. Goodman, Sunjoo Kang

**Affiliations:** ^1^Department of Health Management and Policy, Master of Public Health Program, College of Health and Human Services, University of New Hampshire, Durham, NH, United States; ^2^Faculty of Pharmacy, Universiti Sultan Zainal Abidin, Kuala Terengganu, Terengganu, Malaysia; ^3^Faculty of Pharmacy, Jinnah University for Women, Karachi, Pakistan; ^4^Department of Biostatistics, School of Global Public Health, New York University, New York, NY, United States; ^5^Department of Global Health and Disease Control, Master of Infectious Disease Control Program, Graduate School of Public Health, Yonsei University, Seoul, Republic of Korea

**Keywords:** women in science, public health, education, health promotion, research

To highlight the significant contributions of women to fulfilling the public health mission, this Research Topic features scholarly contributions in the field of Public Health Education and Promotion. This theme is intentionally broad in scope, and sought varied research contributions initiated by women. This editorial provides an overview of the key findings of the papers published in the Research Topic on “*Women in science—Public Health Education and Promotion 2022*.” The types of articles received in response to this Research Topic are summarized below.

## 1. Original research

Cannabis use has been shown to increase the risk of health issues and social problems (1, 2). Kvillemo et al. examined young adults' attitudes toward cannabis use and prevention in Sweden. Participants were recruited through purposeful sampling, and semi-structured, in-depth interviews were conducted using a video platform. The experience of risks with cannabis use varied among the informants who based their risk perception on knowledge received from teachers, authorities, and media. Multi-component drug prevention programs should be implemented, combining a fact-based focus on risks, and delivered by credible and relatable messengers.

Herbal medicine is the most widely used form of traditional medicine. Although there is insufficient data on the safety of herbal medicine, local herbal products are recommended by healthcare professionals in sub-Saharan African countries during pregnancy (3–5). Wake and Fitie assessed the determinants of herbal medicine utilization among pregnant women in Ethiopia. Data were collected via in-person interviews administered using a semi-structured questionnaire. Pregnant women who did not possess an education beyond primary school were more likely to consume herbal medicine during pregnancy in comparison to study participants who possessed a college-level, or higher form of education. Those study participants who had smaller monthly family incomes were more likely to use herbal medicine during pregnancy compared to those pregnant women who had greater monthly family incomes. The authors recommended that healthcare providers should discuss and create awareness about the benefits and complications of herbal medicine utilization during pregnancy.

In another example of health services education and utilization, Balquis et al. identified the quality and storage status of fixed-dose combination antituberculosis drugs and awareness regarding multi-drug-resistant tuberculosis (MDR-TB) among pharmacy staff in Pakistan. The authors noted that the poor control of TB and MDR-TB incidence were closely related to diagnosis time, supply of TB medication, provision of counseling, and implementation of the national TB program. Furthermore, the majority of the pharmacy staff lacked information regarding MDR-TB, and few TB patients had access to the national TB program. The authors concluded that timely identification of TB patients, quality management, and sustainable logistics on essential medication could decrease the MDR-TB incidence rate.

Other research from Pakistan conducted by Malik et al. examined the perception of female healthcare academicians about gender equity and related barriers by implementing a qualitative study design in health professions education. A semi-structured interview encompassing gender equity as an issue, perceived traits, professional relationship with male colleagues, and representation in leadership positions explored the participants' experiences. The participants discussed the level of support and harassment at the workplace, and salary disparity as barriers inhibiting gender equity. Recommendations included the development of policies to advocate for female recruitment, advancement, and equity in the workplace.

Terrorism-related disasters (TRDs) are a rampant challenge globally and impose an unexpected burden on healthcare services that requires adequate preparedness. Considering the high incidence of TRDs in Pakistan and scarcity of information on its management, this qualitative study was designed to evaluate TRD response and preparedness of physicians. Khilji et al. noted that although physicians were prepared professionally and psychologically for dealing with a TRD, they identified critical barriers for the mitigation of TRDs, including lack of disaster-related curricula, training, and resources.

Accessing healthcare services during emergency situations was further examined in the work by Aljabri and Albinali. The Saudi Red Crescent Authority operates Emergency Medical Services (EMS) via the dedicated call number 997 and “Asefny” mobile application. The authors evaluated public awareness and use of the EMS phone number and compared EMS response times between requests made via the 997 phone number and “Asefny” mobile application during the COVID-19 lockdown. A cross-sectional survey was distributed through online platforms and identified several factors that may affect awareness and use of the EMS number, such as gender, location, nationality, education level, having children, and having a chronic illness. The study also found differences in the modes of requesting EMS, with the use of the “Asefny” mobile application being more prevalent than the 997 number.

Lebni et al. examined women's adherence to COVID-19 health protocols in Iran. Purposeful and snowball sampling were used to reach the participants, and semi-structured, in-person interviews were conducted. The authors recommended that it is essential to have a precise understanding of a society's customs and culture to develop effective, multi-faceted interventions targeting individual, environmental, and social factors.

Kawasaki disease (KD) is an acute, self-limited febrile illness of unknown cause that predominantly affects children <5 years of age and is now recognized as a leading cause of acquired heart disease in children in developed countries (6). Zhang et al. examined medication literacy among Chinese caregivers of discharged children with KD. Factors associated with medication literacy were higher education levels, higher income, and longer duration of hospitalization. Caregivers with shorter duration of hospitalization and lower education levels and income should be targeted for medication literacy improvement. The authors proposed that effective communication between caregivers and healthcare providers, comprehendible education materials, training to improve nursing knowledge, and regular follow-ups are methods to improve medication literacy among KD caregivers.

## 2. Systematic review

Gebeyehu et al. conducted a meta-review to identify the determinants of Ethiopian women's discontinuance of long-acting reversible contraceptives. The side effects were the priority reason for discontinuing long-acting contraceptive methods. Predictive factors on women's decision to stop using contraceptives were service dissatisfaction, and the desire to become pregnant.

Liu et al. conducted a systematic review of evidence relevant to tobacco control to improve the effectiveness of health communication. The authors also developed and evaluated tailored health education messages for tobacco control based on target audience input. The authors concluded that the promotion of public health literacy among international populations could contribute to reducing tobacco use and related health issues.

A third systematic review conducted by Deng et al. examined the use of mesenchymal stem cells (MCS) in the field of orthopedics to determine trends and potential areas for future development. The main areas of MSC research included the source of MSCs, *in vitro* experiments, the differentiation process of MSCs, and use of MSCs in knee disease treatment. The authors noted that potential research areas include tissue engineering and uses in orthopedic disease.

## Conclusion

The research highlighted herein demonstrates the many contributions women are making in the field of public health education and promotion globally and thus, comprises the Women in Public Health Education and Promotion, 2022, collection.

## Author contributions

RC led the planning and writing of the editorial. All authors contributed to the writing and review process for the editorial.
